# Performance Features of a Stationary Stochastic Novikov Engine

**DOI:** 10.3390/e20010052

**Published:** 2018-01-12

**Authors:** Karsten Schwalbe, Karl Heinz Hoffmann

**Affiliations:** Institut für Physik, Technische Universität Chemnitz, 09107 Chemnitz, Germany

**Keywords:** finite time thermodynamics, endoreversible thermodynamics, Novikov engine, heat transport, temperature fluctuations, stochastic Novikov engine

## Abstract

In this article a Novikov engine with fluctuating hot heat bath temperature is presented. Based on this model, the performance measure maximum expected power as well as the corresponding efficiency and entropy production rate is investigated for four different stationary distributions: continuous uniform, normal, triangle, quadratic, and Pareto. It is found that the performance measures increase monotonously with increasing expectation value and increasing standard deviation of the distributions. Additionally, we show that the distribution has only little influence on the performance measures for small standard deviations. For larger values of the standard deviation, the performance measures in the case of the Pareto distribution are significantly different compared to the other distributions. These observations are explained by a comparison of the Taylor expansions in terms of the distributions’ standard deviations. For the considered symmetric distributions, an extension of the well known Curzon–Ahlborn efficiency to a stochastic Novikov engine is given.

## 1. Introduction

Power stations that use heat to produce electricity can be understood in principle as heat engines receiving heat from a hot reservoir with temperature $T_{H}$ and releasing heat to a cold reservoir with temperature $T_{L}$. The difference between those two heat amounts is the usable energy of the engine. If the heat engine is fully reversible, the efficiency of the heat engine is the well known Carnot efficiency $\eta_{C}=1-\frac{T_{L}}{T_{H}}$. However, reversibility means infinitely slow processes leading to vanishing power output. Thus, irreversibilities have to be considered to get more realistic formulas for the efficiency of heat engines. Such considerations have been discussed for some time [1,2].

In 1957, the Russian scientist Novikov took those irreversibilities in his model of a heat engine into account by considering a linear heat transport law between the hot heat reservoir and the energy transformation process [3,4]. In this way, he could derive an expression for the efficiency, $\eta_{\mathrm{CA}}=1-\sqrt{\frac{T_{L}}{T_{H}}}$, that turned out to be more realistic than the Carnot one. This term is often referred to as Curzon–Ahlborn efficiency, as Curzon and Ahlborn got the same expression for a slightly different irreversible engine a few years later [5]. These pioneer articles led to *Endoreversible Thermodynamics* [6,7,8]. It is part of the Finite Time Thermodynamics and makes the modelling assumption that all systems can be subdivided into reversible subsystems and, potentially irreversible, interactions between these subsystems. Endoreversible models have been used in the past decades to investigate a large variety of systems [8,9,10,11,12,13,14,15,16,17,18,19,20,21,22,23] with ongoing research in this field [24,25,26,27,28,29]. One of the advantages of Endoreversible Thermodynamics is its ability to model also transient phenomena in thermal processes [6]. Including such transient phenomena has been shown to be important for instance in the evaluation of renewable energy solutions [30].

In their calculations, Novikov as well as Curzon and Ahlborn used constant heat bath temperatures and did not take into account any transient phenomena. While this assumption often seems reasonable, some power stations are characterized by fluctuations of the heat source. For example, in a solar power plant, the changing cloud coverage leads to fluctuations in the intensity of the solar beams, and thus in the temperature of the heat supply. In such an engine, the temperature of the hot heat bath $T_{H}$ will fluctuate.

The consequences of fluctuating temperature will of course be different for different probability density functions of the fluctuating $T_{H}.$ We will thus analyze a sequence of four different distributions functions, which are selected for demonstrating the differences in the performance measures. Hence, we will be able to answer questions like “How does the expectation value and the standard deviation of the hot temperature influence the performance measures?” and “What are the differences in the performance measures for different distribution shapes of the hot temperature?”.

The paper is organized as follows. In Section 2, the classical Novikov engine is presented and afterwards modified by a stochastic component, leading to the stochastic Novikov engine. The expressions for the performance measure maximum power output as well as the corresponding efficiency and entropy production rate are derived. This section is finished by the presentation of an reference example for the further numerical investigations. Thereafter, several temperature distributions are given and the performance measure expressions are evaluated and discussed for these distributions in Section 3. A numerical and analytical comparison is performed and a generalization of the Curzon–Ahlborn efficiency is found. Finally, a conclusion is drawn.

## 2. Novikov Engine with Fluctuating Temperature of the Hot Bath

### 2.1. Classical Novikov Engine

**Figure 1:**
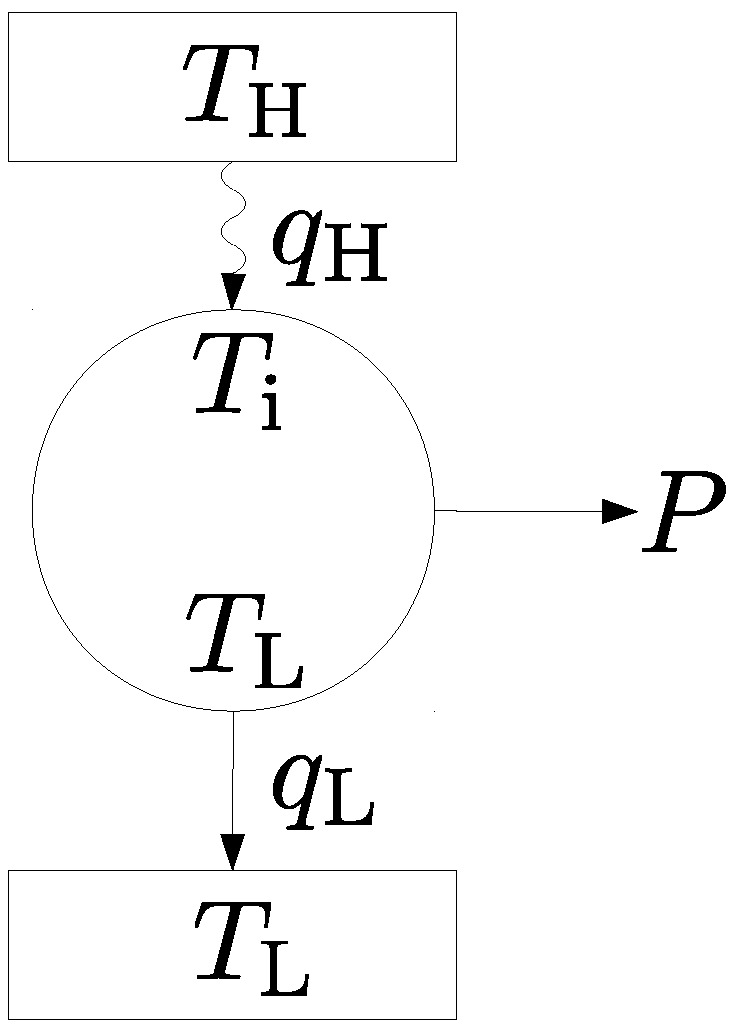
Scheme of a Novikov engine. It consists of a reversible Carnot engine coupled to two heat baths. The heat transport from the hot reservoir to the engine is irreversible.

We start our considerations with a short recapitulation of the classical Novikov engine with constant heat bath temperatures to the extent needed for our considerations. From the Endoreversible Thermodynamics point of view, the Novikov engine can be considered as a reversible Carnot engine working in a steady state mode and transforming an incoming heat flux $q_{H}$ into usable power *P* and an additional heat flux $q_{L}$. The heat flux $q_{H}$ comes from a heat bath with constant temperature $T_{H}$ and enters the engine with temperature $T_{i}$. The heat flux $q_{L}$ leaves the engine with the same constant temperature $T_{L}$ as the cold thermal bath to which the flux is released. Consequently, $q_{H}$ is an irreversible heat flux producing some entropy, while $q_{L}$ is reversible. Usually, the inequality $T_{L}\leq T_{i}\leq T_{H}$ holds true, so $T_{i}$ is often named *internal temperature.* The scheme of the Novikov engine is shown in Figure 1.

For the heat transport between the hot reservoir and the engine Newtonian heat transport is assumed:

(1) $$q_{H}=\kappa \left(T_{H}-T_{i}\right).$$

Here, κ is a proportionality constant including the area of heat transport. Using the energy and entropy balance of the internal Carnot engine,

(2) $$\begin{array}{ccc} q_{H} & = & P+q_{L}, \end{array}$$

(3) $$\begin{array}{ccc} \frac{q_{H}}{T_{i}} & = & \frac{q_{L}}{T_{L}}, \end{array}$$

it follows that

(4) $$P=\kappa \left(T_{H}-T_{i}\right)\left(1-\frac{T_{L}}{T_{i}}\right).$$

We stress that in this model the power reacts instantaneously to a change in $T_{i}$ and thus transient phenomena with a memory are not modeled. Taking κ, $T_{H}$, and $T_{L}$ as fixed, *P* reaches its maximum for

(5) $${\hat{T}}_{i}=\sqrt{T_{H}T_{L}}.$$

At this temperature, the power output is

(6) $$\hat{P}=\kappa {\left(\sqrt{T_{H}}-\sqrt{T_{L}}\right)}^{2}.$$

Note that the values of quantities corresponding to the maximum power operating point will be marked by a hat over the corresponding variable. The efficiency $\eta =\frac{P}{q_{H}}$ at maximum power is

(7) $$\hat{\eta }=1-\sqrt{\frac{T_{L}}{T_{H}}},$$

which is known as Curzon–Ahlborn efficiency [5]. The entropy production $\sigma =q_{H}\left(\frac{1}{T_{i}}-\frac{1}{T_{H}}\right)$ at the maximum power operating point is

(8) $$\hat{\sigma }=\kappa \frac{{\left(\sqrt{T_{H}}-\sqrt{T_{L}}\right)}^{2}}{\sqrt{T_{H}T_{L}}}.$$

$\hat{P}$, $\hat{\eta }$ and $\hat{\sigma }$ play an important role in characterizing heat engines and are thus considered below as the performance measures of interest for these engines.

### 2.2. Stochastic Novikov Engine

While in the classical Novikov engine model the hot temperature $T_{H}$ is assumed to be fixed, it is allowed to fluctuate in the stochastic Novikov engine. For the level of description chosen here, we assume that a stationary probability density function $\rho \left(T_{H}\right)$ is given, which describes the fluctuations of the hot temperature $T_{H}$.

The performance measures of interest are the expected power output $\langle P\rangle$, the expected efficiency $\eta =\frac{\langle P\rangle }{\langle q_{H}\rangle }$, and the expected entropy production $\langle \sigma \rangle$. Here, the common notation $\langle g\rangle$ for the expectation value of an expression *g* with respect to a distribution $\rho (T_{H})$ is used:

(9) $$\langle g\rangle =\int_{0}^{\infty }g\left(T_{H}\right)\rho \left(T_{H}\right)dT_{H}.$$

To ease notation, the brackets at *P*, $q_{H}$ and σ are are now skipped. In the following, the maximum power output $\hat{P}$ and the corresponding efficiency $\hat{\eta }$ as well as the entropy production at maximum power $\hat{\sigma }$ are considered. $\hat{P}$ can be computed using standard analysis methods in the so called feedback control case, where the control variable $T_{i}$ depends on $T_{H}$. Analogously to Equation (5), at the maximum power operating point,

(10) $${\hat{T}}_{i}\left(T_{H}\right)=\sqrt{T_{L}}\sqrt{T_{H}}$$

holds true, i.e., $T_{i}$ is chosen always optimally for the present $T_{H}$. The resulting maximum expected power output is

(11) $$\begin{array}{ccc} \hat{P} & = & \kappa \left(\langle T_{H}\rangle -2\sqrt{T_{L}}\langle \sqrt{T_{H}}\rangle +T_{L}\right) \end{array}$$

with the corresponding efficiency

(12) $$\hat{\eta }=\frac{\langle T_{H}\rangle -2\sqrt{T_{L}}\langle \sqrt{T_{H}}\rangle +T_{L}}{\langle T_{H}\rangle -\sqrt{T_{L}}\langle \sqrt{T_{H}}\rangle }.$$

Finally, we get an entropy production rate

(13) $$\hat{\sigma }=\kappa \left(\frac{\langle \sqrt{T_{H}}\rangle }{\sqrt{T_{L}}}+\sqrt{T_{L}}\langle \frac{1}{\sqrt{T_{H}}}\rangle -2\right).$$

From these equations, we see that $\hat{P}$, $\hat{\eta }$ and $\hat{\sigma }$ depend on the terms $\langle T_{H}\rangle$, $\langle \sqrt{T_{H}}\rangle$, and $\langle \frac{1}{\sqrt{T_{H}}}\rangle$. The first term is just the expectation value of $T_{H}$. The latter two terms depend on the shape of the distribution function, and below we will investigate this dependence in particular in terms of two characteristic values of the distribution function: the expectation value $\langle T_{H}\rangle$ and standard deviation $s=\sqrt{\langle {\left(T_{H}-\langle T_{H}\rangle \right)}^{2}\rangle }$.

### 2.3. Reference Example

**Figure 2:**
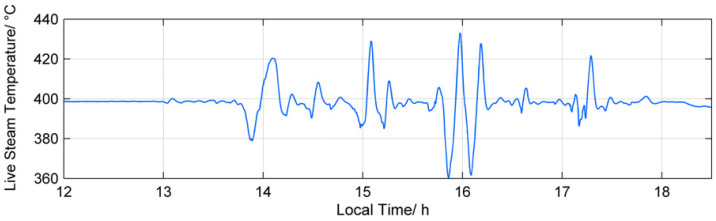
Fluctuating steam temperature as a function of time for a solar power plant, taken from [31]. Note that the temperature can vary by nearly as much as 80 K.

To illustrate our findings and to give the reader a better grasp of the impact of fluctuating parameters on the performance of a heat engine, we will use a solar power plant [31] with direct steam generation as a reference example. Changing cloud cover typically leads to significant changes in the direct normal irradiation, influencing the hot steam temperature of the turbine [31]. These temperature fluctuations are shown in Figure 2. Below, we use the data from that work as a basis for our reference example:

1. The reference example is a heat engine working between a hot bath with temperature $T_{H}$ and a cold heat bath with temperature $T_{L}$.
2. $T_{H}$ has a mean value of 670K.
3. $T_{L}$ has a fixed value of 300K.
4. $\kappa =0.68\;\frac{\mathrm{MW}}{K}$, so the power output is approximately 50MW at $T_{H}=670\;K$.

## 3. Influence of the Temperature Distribution

### 3.1. The Considered Distributions

As already mentioned, the performance measures Equations (11)–(13) depend on the shape of the distribution $\rho (T_{H})$. Here, the analyzed distributions are the uniform, the triangle, the quadratic and the Pareto. The reasons for this selection will be given in the following. The uniform distribution can be used if nothing is known except the range of a stochastic fluctuating quantity. Thus, consequently, it is often assumed that all values inside a certain range are equally likely. In other cases, values at the boundary of a certain given interval are less likely than the values in the center. Furthermore, the extreme values have a vanishing probability. A probability density function, that fulfills these requirements and leads to closed analytical solutions for the performance measures, is the triangle distribution. For many applications, unknown distributions are often assumed to be normal for various reasons. However, the normal distribution has the disadvantage that expressions like $\langle x^{\nu }\rangle$, that will be needed here, have no closed analytic form for ν∉N in general. This problem can be solved by approximating the function around its expectation value by a polynomial via Taylor expansion. As the expectation value is also the modal value in the case of the Normal distribution, the first derivative vanishes and at least a second order approximation has to be made. This motivates the form of the third distribution, the quadratic one. Until now, all the presented distributions are symmetric. In order to investigate the changes occurring if the distribution is non-symmetric, we used here the Pareto distribution as an example of a non-symmetric function.

**Figure 3:**
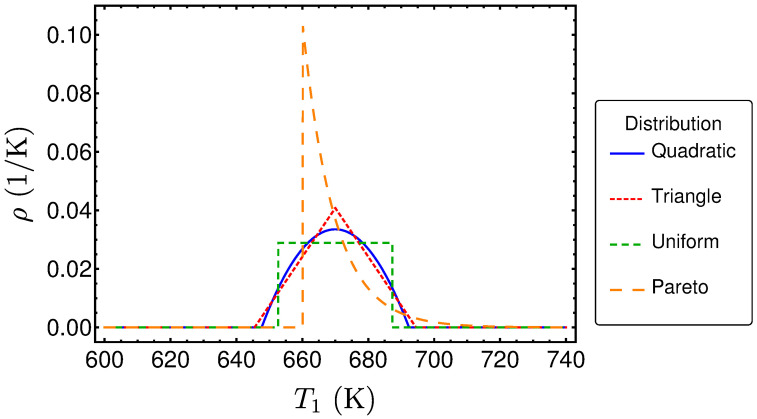
Several distributions ρ as a function of $T_{H}$.

These four distributions are plotted in Figure 3. In order to make them comparable, the same expectation value $\langle T_{H}\rangle =670\;K$ and standard deviation s=10 K are chosen.

In the following, we give the probability density functions for the four considered distributions as they are needed to evaluate the expressions Equations (11)–(13) for the performance measures. We start with the uniform distribution. As already mentioned, this distribution is often chosen when only the minimal value $T_{\min}$ and the maximal value $T_{\max}$ of $T_{H}$ are known. In such a case, the probability density function can be expressed as

(14) $$\rho \left(T_{H}\right)=\left\{\begin{array}{cc} \frac{1}{T_{\max}-T_{\min}}, & \mathrm{for}T_{\min}\leq T_{H}\leq T_{\max},\\ 0, & \mathrm{else}. \end{array}\right.$$

Note that $T_{\min}$ should be greater than $T_{L}$ to guarantee the relation $T_{L}<T_{H}$. Simple calculations lead to the expectation value $\langle T_{H}\rangle =\frac{T_{\min}+T_{\max}}{2}$ and standard deviation $s=\frac{T_{\max}-T_{\min}}{2\sqrt{3}}.$ Using these equations, we can eliminate $T_{\min}$ and $T_{\max}$ from Equation (14), leading to

(15) $$\rho \left(T_{H}\right)=\left\{\begin{array}{cc} \frac{1}{2\sqrt{3}s}, & \mathrm{for}\langle T_{H}\rangle -\sqrt{3}s\leq T_{H}\leq \langle T_{H}\rangle +\sqrt{3}s,\\ 0, & \mathrm{else}. \end{array}\right.$$

Note that obviously $\langle T_{H}\rangle >\sqrt{3}s$ must hold true to guarantee a positive $T_{\min}$.

The other distributions will all be given in terms of $\langle T_{H}\rangle$ and *s*. This makes comparison between deterministic and stochastic results easier, as the deterministic case can be obtained by sending the standard deviation to zero. Additionally, expectation value and standard deviation exist for almost all commonly used distributions, so setting those to fixed values allows one to compare different distributions.

Thus, the probability density of the triangle function can be written as

(16) $$\rho \left(T_{H}\right)=\left\{\begin{array}{cc} \frac{1}{6s^{2}}\left(T_{H}-\langle T_{H}\rangle +\sqrt{6}s\right), & \mathrm{for}\langle T_{H}\rangle -\sqrt{6}s\leq T_{H}\leq \langle T_{H}\rangle ,\\ \frac{1}{6s^{2}}\left(\langle T_{H}\rangle +\sqrt{6}s-T_{H}\right), & \mathrm{for}\langle T_{H}\rangle <T_{H}\leq \langle T_{H}\rangle +\sqrt{6}s,\\ 0, & \mathrm{else}. \end{array}\right..$$

The quadratic distribution can be expressed by

(17) $$\rho \left(T_{H}\right)=\left\{\begin{array}{cc} \frac{-3}{20\sqrt{5}s^{3}}{\left(T_{H}-\langle T_{H}\rangle \right)}^{2}+\frac{3}{4\sqrt{5}s}, & \mathrm{for}\langle T_{H}\rangle -\sqrt{5}s\leq T_{H}\leq \langle T_{H}\rangle +\sqrt{5}s,\\ 0, & \mathrm{else}. \end{array}\right..$$

Finally, the Pareto distribution is given by:

(18) $$\rho \left(T_{H}\right)=\left\{\begin{array}{cc} \frac{\alpha T_{\min}^{\alpha }}{T_{H}^{\alpha +1}}, & T_{H}\geq T_{\min},\\ 0, & \mathrm{else}. \end{array}\right.$$

with

(19) $$\begin{array}{ccc} \alpha & = & 1+\sqrt{{\left(\frac{\langle T_{H}\rangle }{s}\right)}^{2}+1}, \end{array}$$

(20) $$\begin{array}{ccc} T_{\min} & = & \langle T_{H}\rangle +\frac{s^{2}}{\langle T_{H}\rangle }\left(1-\sqrt{{\left(\frac{\langle T_{H}\rangle }{s}\right)}^{2}+1}\right). \end{array}$$

### 3.2. Performances Measures for the Uniform Distribution

Now, the performance measures at maximum power in the case of the uniform distribution are investigated. The relevant expression to calculate the expected power output is $\langle \sqrt{T_{H}}\rangle$:

(21) $$\langle \sqrt{T_{H}}\rangle =\frac{{\left(\langle T_{H}\rangle +\sqrt{3}s\right)}^{\frac{3}{2}}-{\left(\langle T_{H}\rangle -\sqrt{3}s\right)}^{\frac{3}{2}}}{3\sqrt{3}s}.$$

Thus, the maximum expected power is

(22) $$\hat{P}=\kappa \left(\langle T_{H}\rangle -2\sqrt{T_{L}}\frac{{\left(\langle T_{H}\rangle +\sqrt{3}s\right)}^{\frac{3}{2}}-{\left(\langle T_{H}\rangle -\sqrt{3}s\right)}^{\frac{3}{2}}}{3\sqrt{3}s}+T_{L}\right),$$

which in the limit of s→0 gives the classical Novikov result.

**Figure 4:**
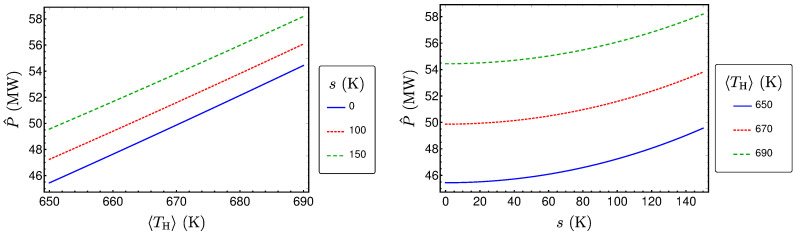
$\hat{P}$ as a function of $\langle T_{H}\rangle$ and *s* for a Novikov engine with Newtonian heat transport and uniformly distributed $T_{H}$.

$\hat{P}$ is plotted in Figure 4 as a function of $\langle T_{H}\rangle$ and *s*. From the figure, we see that $\hat{P}$ increases monotonically with both $\langle T_{H}\rangle$ and *s*, when the other variables are fixed. This behavior can be proven by using standard analysis methods. Thus, consequently, a higher expectation value of the temperature of the hot heat bath results in a higher power output. Additionally, stronger fluctuations (that mean a higher standard deviation) also leads to a higher power output.

The corresponding efficiency at maximum power is

(23) $$\hat{\eta }=\frac{\langle T_{H}\rangle -2\sqrt{T_{L}}\frac{{\left(\langle T_{H}\rangle +\sqrt{3}s\right)}^{\frac{3}{2}}-{\left(\langle T_{H}\rangle -\sqrt{3}s\right)}^{\frac{3}{2}}}{3\sqrt{3}s}+T_{L}}{\langle T_{H}\rangle -\sqrt{T_{L}}\frac{{\left(\langle T_{H}\rangle +\sqrt{3}s\right)}^{\frac{3}{2}}-{\left(\langle T_{H}\rangle -\sqrt{3}s\right)}^{\frac{3}{2}}}{3\sqrt{3}s}}.$$

**Figure 5:**
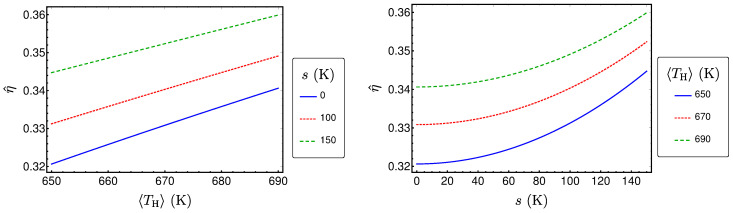
$\hat{\eta }$ as a function of $\langle T_{H}\rangle$ and *s* for a Novikov engine with Newtonian heat transport and uniformly distributed $T_{H}$.

It is plotted in Figure 5 for the reference example. The figure shows that it also increases monotonically with $\langle T_{H}\rangle$ and *s*, respectively. An interesting feature of the produced power as well as of the efficiency is that the relative increase of both is small even for large fluctuations. This is routed in the specific dependence of the power and the efficiency on the hot bath temperature.

**Figure 6:**
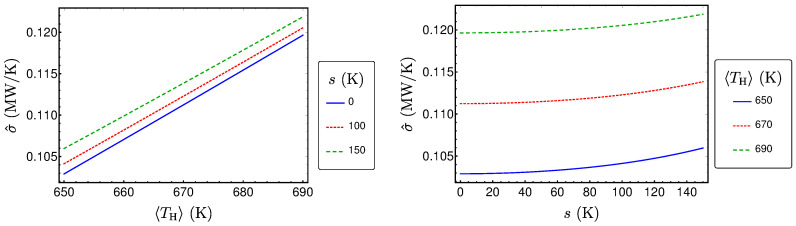
$\hat{\sigma }$ as a function of $\langle T_{H}\rangle$ and *s* for a Novikov engine with Newtonian heat transport and uniformly distributed $T_{H}$.

Finally, $\hat{\sigma }$ is investigated. Using

(24) $$\langle \frac{1}{\sqrt{T_{H}}}\rangle =\frac{2}{\sqrt{\langle T_{H}\rangle -\sqrt{3}s}+\sqrt{\langle T_{H}\rangle +\sqrt{3}s}},$$

and Equation (21), the expected entropy production at maximum power $\hat{\sigma }$ can be calculated according to Equation (13). $\hat{\sigma }$ shows a similar behavior like $\hat{P}$ and $\hat{\eta }$, as it can be seen in Figure 6.

To sum up, all three considered performance measures increase with increasing expectation value and standard deviation, respectively. Here, we have the finding (which is sometimes considered as counterintuitive) that the entropy production rate (“loss”) also increases even though the power output and the efficiency are also increasing.

### 3.3. Comparison of the Performance Measures for Different Distribution Shapes

**Figure 7:**
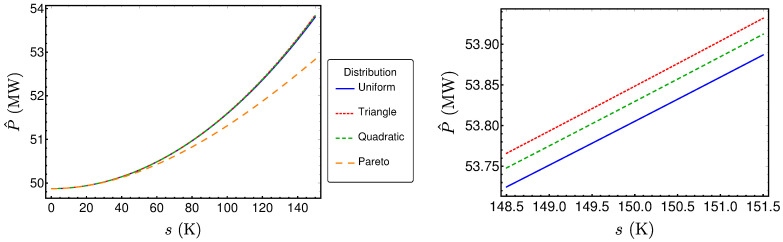
$\hat{P}$ as a function of *s* for different distributions.

**Figure 8:**
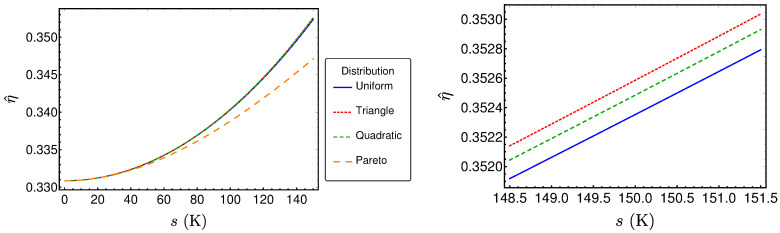
$\hat{\eta }$ as a function of *s* for different distributions.

**Figure 9:**
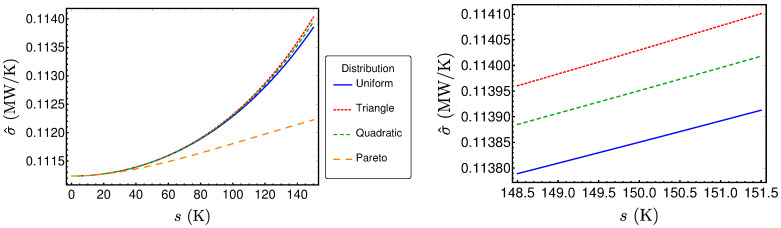
$\hat{\sigma }$ as a function of *s* for different distributions.

The resulting performance measures for the three other distributions can be determined in the same fashion and lead to lengthy expressions similar to those already presented. The results are shown in Figure 7, Figure 8 and Figure 9. Surprisingly, these results are very close together, as one can see for example in Figure 7, where the power output $\hat{P}$ is plotted for the reference example ($\langle T_{H}\rangle =670\;K$) for the different distribution types. The left diagram reveals that the values are very close together for small standard deviations *s*. The only distribution showing larger differences for larger standard deviations is the Pareto distribution, which is the only asymmetric one. However, a closer look on the three symmetric distributions (right diagram) shows that there are small differences in $\hat{P}$ between these distributions. The same holds true for the efficiency $\hat{\eta }$ and $\hat{\sigma }$ (see Figure 8 and Figure 9). In the following, the reason for the closeness of the performance measures will be investigated.

Motivated by the fact that for small *s* the differences of the performance measures seem to be very small, the performance measures are expressed by a Taylor series in *s* for $s\ll \langle T_{H}\rangle$. First, $\frac{\hat{P}}{\kappa }$ is considered. In Table 1, the Taylor coefficients until degree 4 are shown. The term of degree 0 is the result for the classical Novikov engine Equation (6). For all four considered distributions, there is no linear term. Additionally, the coefficients of the quadratic terms are also equal. For the cubic term, first differences occur: while the symmetric distributions do not have it at all, the Pareto distribution has a coefficient different from zero. Finally, the coefficient belonging to degree 4 differs for all four considered distributions.

Thus, the Taylor expansion explains why the values of $\hat{P}$ are very similar for small *s* and why the largest derivations emerge for the Pareto distribution. The sign of the cubic coefficient explains why the value for the Pareto distribution is smaller than the respective values for the symmetric distributions. As $\frac{9}{64}<\frac{75}{448}<\frac{3}{16},$ the power output $\hat{P}$ in the case of a triangle distribution is greater than $\hat{P}$ in the case of a quadratic distribution, and the latter is greater than $\hat{P}$ in the case of a uniform distribution.

Now, the Taylor series of $\hat{\eta }$ is considered (see Table 2). As expected, the term of degree 0 is the Curzon–Ahlborn efficiency. The three symmetric distributions start to differ from the term of degree 4. However, the Pareto distribution has different coefficients starting from the quadratic term. Based on these findings, in the case of uniform, triangle and quadratic distribution for $T_{H}$, the efficiency at maximum power for a Novikov engine with Newtonian heat law can be written as

(25) $$\hat{\eta }=1-\sqrt{\frac{T_{L}}{\langle T_{H}\rangle }}+\frac{T_{L}+\sqrt{T_{L}\langle T_{H}\rangle }}{8\left({\langle T_{H}\rangle }^{3}-\sqrt{T_{L}{\langle T_{H}\rangle }^{5}}\right)}s^{2}+O\left(s^{4}\right).$$

This can be seen as an extension of the original Curzon–Ahlborn efficiency to cases where $T_{H}$ fluctuates stochastically. The term $1-\sqrt{\frac{T_{L}}{\langle T_{H}\rangle }}$ is the Curzon–Ahlborn equivalent, where $T_{H}$ is replaced by its expectation value. Analyzing the second term, one finds that it is always positive, as $\langle T_{H}\rangle \geq T_{\min}>T_{L}$. Consequently, the efficiency at maximum power for some fluctuations in $T_{H}$ is always greater then in the case of fixed $T_{H}=\langle T_{H}\rangle$, at least for those *s* where Equation (25) is a good approximation for the efficiency. Further analysis reveals that

(26) $$\frac{T_{L}+\sqrt{T_{L}\langle T_{H}\rangle }}{8\left({\langle T_{H}\rangle }^{3}-\sqrt{T_{L}{\langle T_{H}\rangle }^{5}}\right)}s^{2}=\frac{1}{8}\sqrt{\frac{T_{L}}{\langle T_{H}\rangle }}\left(\frac{1+\sqrt{\frac{T_{L}}{\langle T_{H}\rangle }}}{1-\sqrt{\frac{T_{L}}{\langle T_{H}\rangle }}}\right){\left(\frac{s}{\langle T_{H}\rangle }\right)}^{2}.$$

Thus, the difference between the efficiencies in the stochastic and in the classical case is greater, when $\sqrt{\frac{T_{L}}{\langle T_{H}\rangle }}$ or $\frac{s}{\langle T_{H}\rangle }$ is larger. This means that high temperatures $T_{L}$ and high fluctuations strengths *s*, relative to the expectation value $\langle T_{H}\rangle$, lead to a high difference in the efficiencies.

For the entropy production $\hat{\sigma }$, the Taylor expansion shows a similar behavior like for the power output $\hat{P}$, as can be seen in Table 3. These terms can be used to estimate $\hat{\sigma }$ in the case of small *s*.

The term of degree 0 corresponds with Equation (13), where the hot temperature $T_{H}$ is replaced by its expectation value $\langle T_{H}\rangle$. Again, the first degree terms vanish. Considering the terms of degree 2, we find that they are equal for the considered distributions. Additionally, we see that they are positive for $\langle T_{H}\rangle <3T_{L}$, which is fulfilled in our reference example. In this case, the entropy production grows monotonously with the distribution’s standard deviation, as already observed. Conversely, if $\langle T_{H}\rangle >3T_{L}$ holds true, the entropy decreases with increasing standard deviation. Comparing the terms of degree 3, we find that they are all zero except in the case of the Pareto distribution. In this case, we find that the degree 3 term is negative when the condition $\langle T_{H}\rangle <5T_{L}$ is met. This is true for our reference example, explaining why the entropy production for the Pareto distribution in Figure 9 lies below the other entropy productions. Consequently, if $\langle T_{H}\rangle >5T_{L}$, the entropy production rate for the Pareto distribution would be higher than the other ones. Finally, the terms of degree 4 explain the observed order for the three symmetric distributions in Figure 9. As $\langle T_{H}\rangle <7T_{L}$ holds true for the reference example and $\frac{9}{128}<\frac{75}{898}<\frac{3}{32}$, the triangle distribution results in the highest entropy production rates and the uniform distribution in the lowest. This order would be changed if $\langle T_{H}\rangle >7T_{L}$. To sum up, our analysis revealed that monotony behavior of the entropy production rate as well as the order of its values according to the different distributions depend on the relation between $\langle T_{H}\rangle$ and $T_{L}$.

## 4. Conclusions

In this article, a stochastic Novikov engine was investigated. In this model, the temperature of the hot heat bath is allowed to fluctuate. For that model, the mean power output was maximized. The resulting expressions for the performance measures maximum power output as well as efficiency and entropy production rate at maximum power were analyzed for different stationary fluctuation shapes. It was found that they all increase monotonously with increasing expectation value and standard deviation of the hot temperature.

Then, the performance measures were compared to each other for the different distributions. For small standard deviations, the performance measures are close together. For larger standard deviations, only the Pareto distribution resulted in significantly different values for the performance measures. A Taylor analysis for small standard deviation revealed the reasons for this observation. It is rooted in the fact that the second order expansion coefficients turn out to be the same for all distributions in the case of the produced power and the entropy production rate. Differences between the distributions only show up for coefficients of order 3 and higher. For the efficiency, the second order expansion coefficients are the same for the symmetric distributions, while those for the asymmetric Pareto distribution differ.

Based on these findings, a generalization of the well known Curzon–Ahlborn efficiency could be obtained for the stochastic Novikov engine. This generalization gives an indication of the efficiency of a power plant operating from a hot temperature source with fluctuating temperature. In particular, our results can help to estimate the effects of varying mean and standard deviation of the fluctuating temperature on the efficiency of power plants. An important feature of the power produced is that quite large fluctuations have a relatively small impact. In the future, it should be investigated whether the results can be generalized for arbitrary distributions. Furthermore, the consequences of stochastic fluctuations on other endoreversible systems should be explored, for instance on those including transient phenomena [30].

## Figures and Tables

**Table 1:** Taylor coefficients of $\frac{\hat{P}}{\kappa }$ for small values of *s*.

Degree	0	1	2	3	4
Uniform	${\left(\sqrt{\langle T_{H}\rangle }-\sqrt{T_{L}}\right)}^{2}$	0	$\frac{1}{4}\sqrt{\frac{T_{L}}{{\langle T_{H}\rangle }^{3}}}$	0	$\frac{9}{64}\sqrt{\frac{T_{L}}{{\langle T_{H}\rangle }^{7}}}$
Triangle	${\left(\sqrt{\langle T_{H}\rangle }-\sqrt{T_{L}}\right)}^{2}$	0	$\frac{1}{4}\sqrt{\frac{T_{L}}{{\langle T_{H}\rangle }^{3}}}$	0	$\frac{3}{16}\sqrt{\frac{T_{L}}{{\langle T_{H}\rangle }^{7}}}$
Quadratic	${\left(\sqrt{\langle T_{H}\rangle }-\sqrt{T_{L}}\right)}^{2}$	0	$\frac{1}{4}\sqrt{\frac{T_{L}}{{\langle T_{H}\rangle }^{3}}}$	0	$\frac{75}{448}\sqrt{\frac{T_{L}}{{\langle T_{H}\rangle }^{7}}}$
Pareto	${\left(\sqrt{\langle T_{H}\rangle }-\sqrt{T_{L}}\right)}^{2}$	0	$\frac{1}{4}\sqrt{\frac{T_{L}}{{\langle T_{H}\rangle }^{3}}}$	$-\frac{1}{4}\sqrt{\frac{T_{L}}{{\langle T_{H}\rangle }^{5}}}$	$-\frac{3}{64}\sqrt{\frac{T_{L}}{{\langle T_{H}\rangle }^{7}}}$

**Table 2:** Taylor coefficients of $\hat{\eta }$ for small values of *s*. The asterisk (*) indicates that the expression is to lengthy to be shown here.

Degree	0	1	2	3	4
Uniform	$1-\sqrt{\frac{T_{L}}{\langle T_{H}\rangle }}$	0	$\frac{T_{L}+\sqrt{T_{L}\langle T_{H}\rangle }}{8\left({\langle T_{H}\rangle }^{3}-\sqrt{T_{L}{\langle T_{H}\rangle }^{5}}\right)}$	0	$\frac{9\sqrt{T_{L}}\langle T_{H}\rangle -2T_{L}\sqrt{\langle T_{H}\rangle }-11T_{L}^{3/2}}{128{\langle T_{H}\rangle }^{9/2}\left(T_{L}+\langle T_{H}\rangle -2\sqrt{T_{L}\langle T_{H}\rangle }\right)}$
Triangle	$1-\sqrt{\frac{T_{L}}{\langle T_{H}\rangle }}$	0	$\frac{T_{L}+\sqrt{T_{L}\langle T_{H}\rangle }}{8\left({\langle T_{H}\rangle }^{3}-\sqrt{T_{L}{\langle T_{H}\rangle }^{5}}\right)}$	0	$\frac{6\sqrt{T_{L}}\langle T_{H}\rangle -T_{L}\sqrt{\langle T_{H}\rangle }-7T_{L}^{3/2}}{64{\langle T_{H}\rangle }^{9/2}\left(T_{L}+\langle T_{H}\rangle -2\sqrt{T_{L}\langle T_{H}\rangle }\right)}$
Quadratic	$1-\sqrt{\frac{T_{L}}{\langle T_{H}\rangle }}$	0	$\frac{T_{L}+\sqrt{T_{L}\langle T_{H}\rangle }}{8\left({\langle T_{H}\rangle }^{3}-\sqrt{T_{L}{\langle T_{H}\rangle }^{5}}\right)}$	0	$\frac{75\sqrt{T_{L}}\langle T_{H}\rangle -14T_{L}\sqrt{\langle T_{H}\rangle }-89T_{L}^{3/2}}{896{\langle T_{H}\rangle }^{9/2}\left(T_{L}+\langle T_{H}\rangle -2\sqrt{T_{L}\langle T_{H}\rangle }\right)}$
Pareto	$1-\sqrt{\frac{T_{L}}{\langle T_{H}\rangle }}$	0	$\frac{{\langle T_{H}\rangle }^{2}\left(T_{L}^{2}{\langle T_{H}\rangle }^{3}+\sqrt{T_{L}{\langle T_{H}\rangle }^{9}}-T_{L}\left({\langle T_{H}\rangle }^{4}+\sqrt{T_{L}{\langle T_{H}\rangle }^{7}}\right)\right)}{8{\left({\langle T_{H}\rangle }^{3}-\sqrt{T_{L}{\langle T_{H}\rangle }^{5}}\right)}^{3}}$	*	*

**Table 3:** Taylor coefficients of $\frac{\hat{\sigma }}{\kappa }$ for small values of *s*.

Degree	0	1	2	3	4
Uniform	$\frac{{\left(\sqrt{\langle T_{H}\rangle }-\sqrt{T_{L}}\right)}^{2}}{\sqrt{\langle T_{H}\rangle T_{L}}}$	0	$\frac{3T_{L}-\langle T_{H}\rangle }{8\sqrt{T_{L}}{\langle T_{H}\rangle }^{5/2}}$	0	$\frac{9\left(7T_{L}-\langle T_{H}\rangle \right)}{128\sqrt{T_{L}}{\langle T_{H}\rangle }^{9/2}}$
Triangle	$\frac{{\left(\sqrt{\langle T_{H}\rangle }-\sqrt{T_{L}}\right)}^{2}}{\sqrt{\langle T_{H}\rangle T_{L}}}$	0	$\frac{3T_{L}-\langle T_{H}\rangle }{8\sqrt{T_{L}}{\langle T_{H}\rangle }^{5/2}}$	0	$\frac{3\left(7T_{L}-\langle T_{H}\rangle \right)}{32\sqrt{T_{L}}{\langle T_{H}\rangle }^{9/2}}$
Quadratic	$\frac{{\left(\sqrt{\langle T_{H}\rangle }-\sqrt{T_{L}}\right)}^{2}}{\sqrt{\langle T_{H}\rangle T_{L}}}$	0	$\frac{3T_{L}-\langle T_{H}\rangle }{8\sqrt{T_{L}}{\langle T_{H}\rangle }^{5/2}}$	0	$\frac{75\left(7T_{L}-\langle T_{H}\rangle \right)}{896\sqrt{T_{L}}{\langle T_{H}\rangle }^{9/2}}$
Pareto	$\frac{{\left(\sqrt{\langle T_{H}\rangle }-\sqrt{T_{L}}\right)}^{2}}{\sqrt{\langle T_{H}\rangle T_{L}}}$	0	$\frac{3T_{L}-\langle T_{H}\rangle }{8\sqrt{T_{L}}{\langle T_{H}\rangle }^{5/2}}$	$\frac{-5T_{L}+\langle T_{H}\rangle }{8\sqrt{T_{L}}{\langle T_{H}\rangle }^{7/2}}$	$\frac{3\left(25T_{L}+\langle T_{H}\rangle \right)}{128\sqrt{T_{L}}{\langle T_{H}\rangle }^{9/2}}$
